# Search-optimized quantization in biomedical ontology alignment

**DOI:** 10.3389/frai.2025.1662984

**Published:** 2025-10-10

**Authors:** Oussama Bouaggad, Natalia Grabar

**Affiliations:** ^1^CNRS, Univ. Lille, UMR 8163 - STL - Savoirs Textes Langage, Lille, France; ^2^Univ. Lille, UMR 9189 - CRIStAL - Centre de Recherche en Informatique Signal et Automatique de Lille, Lille, France

**Keywords:** UMLS Metathesaurus, ontology alignment, semantic similarity, transformer models, model optimization, model quantization

## Abstract

In the fast-moving world of AI, as organizations and researchers develop more advanced models, they face challenges due to their sheer size and computational demands. Deploying such models on edge devices or in resource-constrained environments adds further challenges related to energy consumption, memory usage and latency. To address these challenges, emerging trends are shaping the future of efficient model optimization techniques. From this premise, by employing supervised state-of-the-art transformer-based models, this research introduces a systematic method for ontology alignment, grounded in cosine-based semantic similarity between a biomedical layman vocabulary and the Unified Medical Language System (UMLS) Metathesaurus. It leverages Microsoft Olive to search for target optimizations among different Execution Providers (EPs) using the ONNX Runtime backend, followed by an assembled process of dynamic quantization employing Intel Neural Compressor and IPEX (Intel Extension for PyTorch). Through our optimization process, we conduct extensive assessments on the two tasks from the DEFT 2020 Evaluation Campaign, achieving a new state-of-the-art in both. We retain performance metrics intact, while attaining an average inference speed-up of 20x and reducing memory usage by 70%.

## 1 Introduction

Biomedical ontology alignment refers to the process of matching semantically related entities across diverse knowledge sources (databases) to facilitate the integration of heterogeneous data. The historical impetus for biomedical ontology alignment arose from the need to consolidate independently developed knowledge sources, each characterized by distinct data vocabularies. In this domain, the Unified Medical Language System (UMLS) Metathesaurus (Bodenreider, 2004), developed under the auspices of the U.S. National Library of Medicine (NLM), serves as a cornerstone.^1^ The UMLS Metathesaurus, which comprises the most extensive collection of biomedical ontologies, including terminologies, controlled vocabularies, thesauri, and classifications, provides an essential framework for unifying standardized knowledge sources. With the ongoing evolution of this project, its size has reached over 10 million atoms, derived from more than 200 controlled vocabularies grouped into approximately 4 million concepts. Its maintenance process is costly, time-consuming, and places significant demands on expert editors. However, decades of meticulous manual curation provide ample material for modern supervised learning applications, establishing UMLS as a foundational resource for ontology alignment. Conversely, the biomedical layman vocabulary (Koptient and Grabar, 2020) is designed to support the adaptation and simplification of medical texts. Its purpose is to enhance understanding of health-related documents for non-expert audiences, such as patients. Its size is steadily increasing, although it remains significantly smaller than that of large-scale terminologies. The alignment of the layman vocabulary with UMLS is important for ensuring that structured medical knowledge is accessible and useful to non-experts, thereby improving the effectiveness of healthcare communication. This helps bridge the language gap between clinicians and patients, allowing for dynamic adjustment of linguistic complexity. Nevertheless, achieving accurate alignment between layman and expert terms presents significant challenges. These include lexical variation, contextual ambiguity, and the frequent absence of direct one-to-one concept mappings. Furthermore, layman expressions often lack the ontological grounding and semantic precision of formal vocabularies, making purely symbolic or rule-based methods inadequate.

Alongside this, advances in Natural Language Processing (NLP), such as entity linking and semantic similarity, are continuously evolving through state-of-the-art transformer-based supervised deep learning models, incorporating feature engineering with specialized domain knowledge. In this contextualized undertaking, we propose using two approaches, the krissbert (Knowledge-RIch Self-Supervision) model developed by Microsoft Research (Zhang et al., 2022) and the large variant of the Sapbert model from Cambridge LTL (Liu et al., 2021) to align the layman vocabulary with UMLS via cosine-based semantic similarity.

Upon generating the vocabulary, the biomedical alignments are manually verified by expert human annotators using a six-point rating scale, ranging from 0 to 5, to assess degrees of similarity (Dagan et al., 2009). Additional semantic information is included by incorporating all Metathesaurus data file domains and their respective hierarchical structures. These are systematically aligned by means of a left join propagation based on the common *CUI (Concept Unique Identifier)* field.

In conjunction with this, model selection is based on the distinct characteristics of each model, as no single transformer is expected to consistently handle all nuanced details and noise in alignments. Hence, a dual-model approach is used, ensuring that inaccuracies from one model are mitigated by the other. To operationalize this complementarity, alignments are merged iteratively in descending order of rating: starting with all alignments rated 5 by one model, followed by those rated 5 by the other model that are not already included, and proceeding through lower-rated alignments until a comprehensive, high-confidence set is constructed. This dualism leverages the complementary strengths of KRISSBERT and SapBERT, ensuring robust performance across diverse biomedical vocabulary contexts. The KRISSBERT model addresses ambiguity and context-ignorance, particularly where entities share similar surface forms, by harnessing contextual information to improve identification accuracy. This is achieved by training a contextual mention encoder using contrastive learning with a transformer-based encoder (Vaswani et al., 2017) and improving linking accuracy by re-ranking the top *K* candidates with a cross-attention encoder (Logeswaran et al., 2019; Wu L. et al., 2020). On the other hand, the large version of SapBERT introduces a pretraining metric learning framework grounded in self-supervised masked language modeling. It learns to self-align synonymous biomedical entities, accurately capturing fine-grained semantic relationships by clustering synonyms under the same concept. It distinguishes itself from existing systems through a streamlined design that eliminates complex hybrid tuning components, directly encoding and aligning medical entities from raw text (Xu et al., 2020; Ji et al., 2020; Sung et al., 2020).

The large scale of the alignment task imposes a significant computational cost, laying the groundwork for a bottleneck. For this reason, we propose an interoperable cutting-edge optimization process focused on quantization. Fundamentally, it is significant to highlight that the performance of the alignment techniques is intricately linked to two major factors: time requirements and computational resource limitations. Accordingly, Microsoft Olive is leveraged to intelligently search for optimizations among different Execution Providers (EPs) using the ONNX Runtime backend. Sequentially, an accuracy-preserving quantization is then applied using Intel Neural Compressor and IPEX, with SmoothQuant (Xiao et al., 2024). This approach shifts quantization complexity from activations to weights. It strategically engineers the scaling factor matrix *S* to parameterize this process, along with the smoothing factor α, in order to mathematically resolve both the dequantization complexity and the inherent incompatibility with modern accelerated hardware computation kernels. The latter requires high efficiency and cannot tolerate lower-throughput operations. To further assess the optimization impact, calibration procedures are systematically conducted using diverse biomedical datasets, specifically aimed at evaluating model performance in aligning terminology across heterogeneous sources.

To rigorously quantify the robustness of our optimization strategies through the trade-off between performance, latency, and resource consumption, we conduct comprehensive evaluations using the huggingface_metrics backend. These are carried out on the two established benchmark tasks from the DEFT 2020 Evaluation Campaign (Cardon et al., 2020), as they closely align with our core research objectives. Our work democratizes the use of deep learning applications by offering a scalable, turnkey solution that significantly reduces serving costs without compromising model accuracy.

## 2 Related work

### 2.1 Biomedical ontology alignment

Since knowledge source builders concerned with developing health systems for various model organisms joined to create the Gene Ontology Consortium in 1998, the need for biomedical ontology alignment applications (Lambrix, 2004) has grown significantly, aiming to determine correspondences between concepts across different ontologies (Euzenat and Shvaiko, 2007). Scalable logic-based ontology matching systems, including LogMap (Jiménez-Ruiz and Cuenca Grau, 2011) and AgreementMakerLight (AML) (Faria et al., 2013), treat alignment as a sequential process, starting with lexical matching, followed by mapping extension and correction. However, these systems primarily consider surface-level text forms, neglecting word semantics.

Recent machine learning approaches, such as DeepAlignment (Kolyvakis et al., 2018) and OntoEmma (Wang L. L. et al., 2018), map words into vector spaces using embeddings, where semantically closer words have smaller similarity distances. Yet, non-contextual embeddings limit their ability to disambiguate meaning. Fine-tuned BERT models (He et al., 2021) and Siamese Neural Networks (SiamNN) (Chen et al., 2021) demonstrate improved performance, but challenges remain due to limited annotated data and the large entity space.

To address these challenges, we adopt ontology alignment systems based on state-of-the-art supervised learning schemes, utilizing domain-specific knowledge from UMLS. Our approach combines KRISSBERT (Zhang et al., 2022), which effectively resolves variations and ambiguities among millions of entities through self-supervision, and the large SapBERT variant (Liu et al., 2021), which employs an extensive metric learning framework to self-align synonymous biomedical entities, linking synonyms into a unified semantic notion. Unlike pragmatic pretrained models, notably Biobert (Lee, Lee et al., 2020), PubMedbert (Gu et al., 2021), and Bioformer (Fang et al., 2023), which still require labeled data such as gold mention occurrences, constrained by annotation scarcity across expansive biomedical domains, and struggle to produce well-differentiated embedding spaces, our approach captures contextual meaning more efficiently. It coherently retrieves all UMLS entities sharing surface forms and supports the generation of distinct representations for semantically different biomedical concepts.

### 2.2 Model optimizations

Techniques for accelerating and compressing deep learning models have garnered significant attention due to their ability to reduce parameters, computations, and energy-intensive memory access. Optimization methods in neural networks date back to the late 1980s (LeCun et al., 1989; Nowlan and Hinton, 1992), with quantization (approximating numerical components with low bit-width precision) (Jacob et al., 2018; Wu H. et al., 2020; Rokh et al., 2023), pruning (removing less important connections to create sparse networks) (Hassibi and Stork, 1992; Frankle and Carbin, 2019), and knowledge distillation (teacher-student neural model paradigm) (Hinton et al., 2015; Xu et al., 2017) becoming widely adopted. These techniques allow smaller models to operate efficiently within energy-saving on-chip memory, reducing reliance on high-latency off-chip DRAM. Recent advances highlight the importance of combining optimization strategies for greater efficiency (Wang et al., 2020; Park et al., 2022). Quantization, achieving significant compression with minimal accuracy loss (Carreira-Perpiñán, 2017), is often paired with pruning (Yu et al., 2020; Qu et al., 2020), automatic mixed precision (Micikevicius et al., 2018; Rakka et al., 2022), and performance tuning (Roy et al., 2023) in sequential pipelines. Extensively applied in transformers (Shen et al., 2020; Kim et al., 2021; Schaefer et al., 2023), quantization benefits from techniques such as weight equalization (Nagel et al., 2019) and channel splitting (Zhao et al., 2019), which address weight outliers but fall short in handling activation outliers, a persistent bottleneck. To solve these challenges, our novel proposed quantization approach mitigates activation outliers by shifting the complexity to weight quantization (Xiao et al., 2024), streamlining computational operations.

### 2.3 End-to-end hardware-aware optimizations

Initially, researchers focused on software-level optimizations before addressing hardware efficiency (Han et al., 2015; Courbariaux et al., 2015). However, such a static approach fails to exploit the full potential of combining diverse compression techniques to improve performance (Guo et al., 2016; Yang et al., 2020). By optimizing memory access patterns and leveraging parallelism, compressed models significantly reduce both hardware costs and computational resource demands (Shivapakash et al., 2020; Huai et al., 2023; Balaskas et al., 2024). To this end, we leverage Microsoft Olive, with its dedicated hardware-aware ecosystem, to systematically engineer and automate the optimization process.

## 3 Methodology

In line with our study objective, which focuses on aligning biomedical ontologies using cosine similarity measures, we align the concatenation of two fields, *Biomedical Term* and *Public Explanation*, from the layman biomedical vocabulary with all the French entries in the *String (ST)* field of the MRCONSO.RRF raw file from the AB2024 UMLS Metathesaurus release. To accomplish this, we devised a sequential algorithmic search process designed to optimize model performance across multiple EPs. It integrates network compression, parallel processing, and memory transfer optimization through Microsoft Olive, in cooperation with the ONNX Runtime backend, thus enabling efficient and scalable execution. Furthermore, within this framework, we employ Intel Neural Compressor and IPEX, incorporating the logic of SmoothQuant, to design a search-optimized, on-the-fly quantization strategy (W8A8). This approach uniformly shifts the burden from activation outliers to weights, thereby enhancing compatibility with specific hardware-accelerated kernels.

By adopting this strategy, memory usage is significantly reduced and inference speed improved, both critical factors for effective alignment. This synergy, essential to the performance of biomedical ontology systems, depends on these optimizations to ensure dynamic scalability.

### 3.1 Formal definition

An ontology is typically defined as an explicit specification of a conceptualization. It often uses representational vocabularies to describe a domain of interest, with the main components being entities^2^ and axioms. Ontology alignment involves matching cross-ontology entities with equivalence, subsumption, or related relationships. Alongside this, the current study focuses on equivalence alignment between classes.^3^

The ontology alignment system inputs a pair of ontologies, *O* and *O*′, with class sets *C* and *C*′. It generates, using cosine similarity, a set of scored mappings in the form (*c* ∈ *C, c*′ ∈ *C*′, *P*(*c* ≡ *c*′)), where *P*(*c* ≡ *c*′) ∈ [0, 1] is the probability score (*mapping value*) of equivalence between *c* and *c*′. Final mappings are selected based on the highest scores, leveraging supervised SOTA learning schemes with feature engineering. When one model produces more accurate alignments, these are used to correct those of the other, with manual verification by human annotators to improve reliability.

In the present architecture, the input sequence includes a special token [CLS], the tokens of two sentences *A* and *B*, and the special token [SEP] separating them. Each token embedding encodes its content, position, and sentence information. In L successive layers of the architecture, the multi-head self-attention block computes contextualized representations for each token. The output of layer *l* is the embedding sequence derived from the input, as defined in Equation 1:


(1) $$\begin{array}{l} f_{bert}(x,l)=(v_{CLS}^{\left(l\right)},v_{1}^{\left(l\right)},\ldots ,v_{N}^{\left(l\right)},\\ \;v_{SEP}^{\left(l\right)},v_{1}^{\prime (l)},\ldots ,v_{N\prime }^{\prime (l)})\\ \;\in {\mathbb{R}}^{(N+N^{\prime }+2)\times d} \end{array}$$

where **x** is the input sequence, ${\text{v}}_{i}^{\left(l\right)}$ and $v_{j}^{\prime (l)}$ are *d*-dimensional vectors of the corresponding tokens. The final layer (l=L) outputs the resulting token embeddings. Unlike non-contextual embeddings such as Word2Vec (Mikolov et al., 2013), which assign one embedding per token, this configuration distinguishes occurrences of the same token in different contexts. This is critical in expanding biomedical domains where traditional embeddings are biased toward frequent meanings in training corpora. For instance, the acronym “MS” can refer to *Multiple Sclerosis*, a chronic neurological disease affecting the central nervous system, or to *Mass Spectrometry*, an analytical technique used to measure ion mass-to-charge ratios in chemical and biological samples.

Concordantly, given input ontologies *O* and *O*′ with class sets *C* and *C*′, a naive algorithm computes alignments by looking up $c^{\prime }=\arg\max_{c^{\prime }\in C^{\prime }}P\left(c\equiv c^{\prime }\right)$ for each *c* ∈ *C*, leading to *O*(*n*^2^) time complexity. This is parametrically enhanced via Microsoft Olive, which employs an algorithmic search approach that calibrates a joint^4^ execution order, backed by the TPE (Tree-structured Parzen Estimator) algorithm.

**Figure 1:**
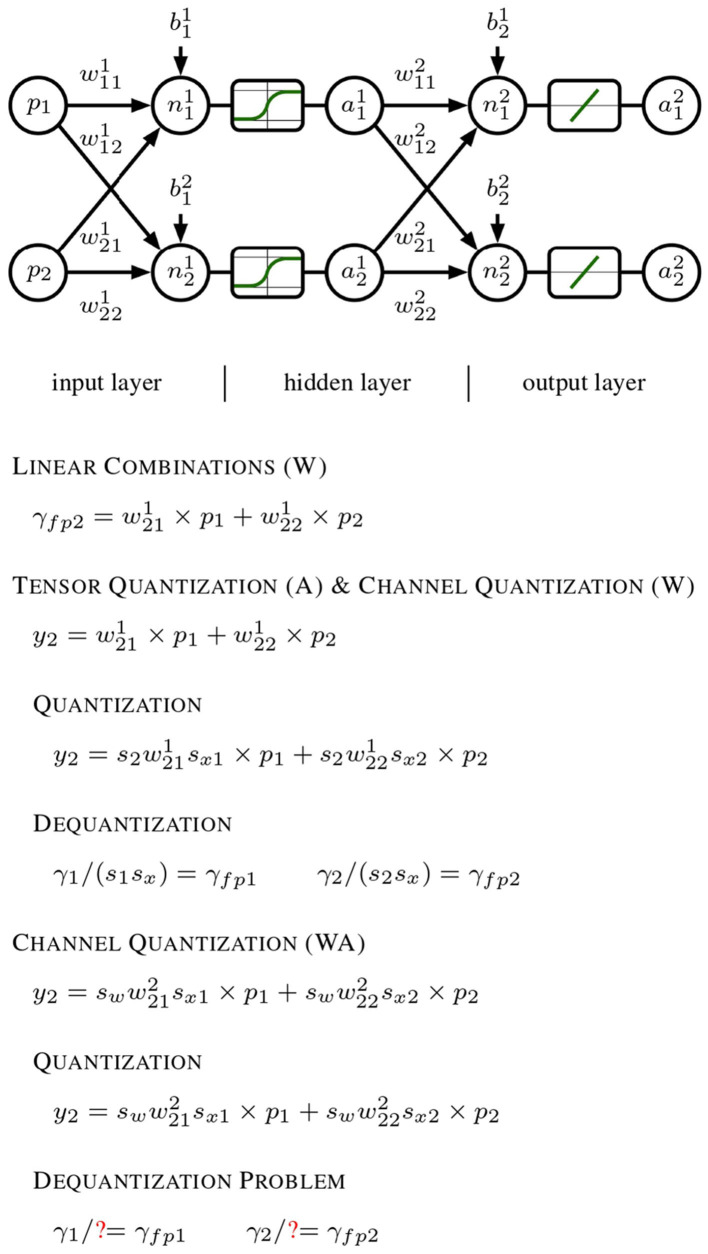
Progression of quantization techniques applied to a generic neural network model. It begins with a linear forward pass using a 1 × 2 input *x* and a 2 × 2 weight matrix *W*, which produces the outputs *y*_1_ and *y*_2_ in a straightforward floating-point manner. In the middle section, per-tensor quantization is performed on activation outputs, and per-channel quantization on weights. The quantized outputs ŷ_1_ and ŷ_2_ can be dequantized to their original floating-point values *y*_*fp*1_ and *y*_*fp*2_ using the channel-specific scales 1.0/(*s*_1_*s*_*x*_) and 1.0/(*s*_2_*s*_*x*_), respectively. Finally, both weights and activations undergo per-channel quantization. This additional layer of complexity hinders accurate dequantization of ŷ_1_ and ŷ_2_ back to their original floating-point results, as the activation quantization depends on the specific channel.

Our search-optimized quantization pipeline (W8A8) further improves efficiency by shifting computational complexity from activations to weights, ensuring seamless integration with hardware-accelerated compute units and resolving^5^ dequantization issues, conforming to Figure 1.

The present failure occurs due to the mathematical incompatibility^6^ between the quantization scales applied to the different channels, which prevents a straightforward dequantization process that would otherwise be possible in the earlier stages with simpler per-tensor and per-channel quantization.

### 3.2 Mathematical model

Following optimization, the dynamically quantized model, along with the tokenizer $T:D\to {\mathbb{R}}^{B\times L\times D}$, is loaded, where *B* is the batch size, *L* is the sequence length, and *D* is the embedding dimension. The domain D denotes the set of raw text inputs.

In turn, a function to batch encode the lists of interest is introduced. It initializes data structures to collect text batch embeddings, while storing intermediate results temporarily to streamline alignment mechanisms. This step ensures that subsequent computations are performed efficiently, improving overall throughput and avoiding memory bottlenecks during batch processing.

The set of texts **T** = {*T*_1_, *T*_2_, …, *T*_*N*_}, with *N* = |**T**|, is divided into batches of size *B* = 10, denoted as **B**_*k*_ for *k* = 1, …, *K*, where $K=\left\lceil \frac{N}{B}\right\rceil$, as formulated in Equation 2:


(2) $$\begin{array}{l} \text{T}=\overset{K}{\underset{k=1}{⋃}}{\text{B}}_{k} \end{array}$$

Each batch **B**_*k*_ is defined as in Equation 3:


(3) $$\begin{array}{l} {\text{B}}_{k}=\left\{T_{\left(k-1\right)B+1},T_{\left(k-1\right)B+2},\ldots ,T_{\min\left(kB,N\right)}\right\} \end{array}$$

Accordingly, the tokenizer T maps the textual input in each batch **B**_*k*_ to its numerical tensor representation **X**_*k*_, as established in Equation 4:


(4) $$\begin{array}{l} {\text{X}}_{k}=T\left({\text{B}}_{k}\right) \end{array}$$

where the tokenized data ${\text{X}}_{k}\in {\mathbb{R}}^{B\times L\times D}$ represents each batch. Thus, padding and truncation ensure uniform sequence lengths, with *L* = 512 set via the max_length parameter. The resulting outputs are converted into PyTorch tensors, enabling consistent formatting across batches. This standardization reinforces compatibility and integration with ONNX-based pipelines, after which the tensors are cast to NumPy arrays for seamless transfer within the processing infrastructure.

ONNX Runtime is then activated by initiating a session that processes the dynamically quantized model $M:{\mathbb{R}}^{B\times L\times D}\to {\mathbb{R}}^{B\times L\times H}$, producing the embeddings **H**_*k*_, given by Equation 5:


(5) $$\begin{array}{l} {\text{H}}_{k}=M\left({\text{X}}_{k}\right) \end{array}$$

where ${\text{H}}_{k}=\left[{\text{h}}_{kij}\right]\in {\mathbb{R}}^{B\times L\times H}$, with ${\text{h}}_{kij}\in {\mathbb{R}}^{H}$ denoting the hidden-state vector corresponding to the *j*-th token of the *i*-th input in batch *k*, and *H* denoting the model's output hidden dimension.

Embeddings are subsequently converted into PyTorch tensors and averaged across the sequence length to produce fixed-size, batch-level representations, in accordance with Equation 6:


(6) $$\begin{array}{l} {\text{e}}_{ki}=\frac{1}{L}\overset{L}{\underset{j=1}{\sum }}{\text{h}}_{kij} \end{array}$$

This yields ${\text{E}}_{k}\in {\mathbb{R}}^{B\times H}$, where each row **e**_*ki*_ corresponds to the mean-pooled embedding of a single input in batch *k*. The final dataset-level embedding matrix **E** ∈ ℝ^*N*×*H*^ is then constructed by stacking all individual embedding vectors ${\text{e}}_{i}^{⊤}\in {\mathbb{R}}^{1\times H}$ (for *i* = 1, …, *N*), which are grouped into the batch-level matrices **E**_*k*_ (for *k* = 1, …, *K*), as detailed in Equation 7:


(7) $$\begin{array}{l} \text{E}=\left[\begin{array}{c} {\text{e}}_{1}^{⊤}\\ {\text{e}}_{2}^{⊤}\\ \vdots \\ {\text{e}}_{N}^{⊤} \end{array}\right]=\left[\begin{array}{c} {\text{E}}_{1}\\ \vdots \\ {\text{E}}_{K} \end{array}\right] \end{array}$$

Using this function, two sets of texts are encoded, as specified in Equation 8, producing the embeddings tensors **E**_*L*_ and **E**_*M*_, where **L** = {*T*_*L*_1__, …, *T*_*L*__*N*__*L*___} and **M** = {*T*_*M*_1__, …, *T*_*M*__*N*__*M*___} are the input collections from LEX and MRCONSO, respectively:


(8) $$\begin{array}{l} \begin{array}{c} {\text{E}}_{L}=\text{EncodeBatch}\left(\text{L}\right)\in {\mathbb{R}}^{N_{L}\times H}\\ {\text{E}}_{M}=\text{EncodeBatch}\left(\text{M}\right)\in {\mathbb{R}}^{N_{M}\times H} \end{array} \end{array}$$

Cosine similarity is then computed to quantify pairwise semantic similarity between embeddings. For two vectors **a** and **b**, it is defined as in Equation 9:


(9) $$\begin{array}{l} \text{cosine\_similarity}\left(\text{a},\text{b}\right)=\frac{{\text{a}}^{⊤}\text{b}}{∥\text{a}{∥}_{2}∥\text{b}{∥}_{2}} \end{array}$$

The resulting matrix $\text{S}\in {\mathbb{R}}^{N_{L}\times N_{M}}$, where each element (*i, j*) represents the similarity between the *i*-th embedding vector ${\text{E}}_{Li}\in {\mathbb{R}}^{H}$ in LEX and the *j*-th embedding vector ${\text{E}}_{Mj}\in {\mathbb{R}}^{H}$ in MRCONSO, is obtained as in Equation 10:


(10) $$\begin{array}{l} {\text{S}}_{ij}=\text{cosine\_similarity}\left({\text{E}}_{Li},{\text{E}}_{Mj}\right)\\ \;=\frac{{\text{E}}_{Li}^{⊤}{\text{E}}_{Mj}}{∥{\text{E}}_{Li}{∥}_{2}∥{\text{E}}_{Mj}{∥}_{2}} \end{array}$$

Finally, each term *T*_*L*_*i*__ in LEX is aligned to its closest semantic counterpart in MRCONSO by selecting the index $j_{i}^{*}$ that maximizes the cosine similarity, as determined in Equation 11:


(11) $$\begin{array}{l} j_{i}^{*}=\arg\underset{j}{\max}{\text{S}}_{ij} \end{array}$$

## 4 Experiments and discussions

### 4.1 Experimental setups

#### 4.1.1 Preprocessing

To achieve this, the dataset of the French layman biomedical lexicon, originally in TXT format, is converted into a DataFrame and defined as LEX. Similarly, the AB2024 version of MRCONSO (extracted by selecting all French entries via MetamorphoSys), originally in RRF format, is also converted into a DataFrame and referred to as MRCONSO. Since the transformer-based models under study are in English, LEX is augmented with the English translations of the fields of interest *Biomedical Term* and *Public Explanation*, using the Google Translate API. The same translation is applied to the *String (ST)* field of MRCONSO. Data integrity is then verified through statistical analysis, assessing distributional properties, missing values, and outliers.

Subsequently, text preprocessing is performed via a multi-step pipeline of cleaning and normalization. This includes converting text to lowercase, removing non-alphanumeric characters, normalizing spaces, removing stopwords, and applying lemmatization through the ScispaCy model (Neumann et al., 2019). The resulting outputs are concatenated into a list format for modular processing.^7^

#### 4.1.2 AI high-performance computing (HPC)

The transformer-based models undergo comprehensive optimization via the infrastructure of Microsoft Olive. This optimization process refines architectural configurations by leveraging symbolic shape inference to understand tensor shapes.

Microsoft Olive is used to explore optimal configurations across ONNX Runtime Execution Providers, specifically CUDAExecutionProvider and TensorRTExecutionProvider. This is achieved using a JSON-based configuration file (olive_config.json) and a custom script (user_script.py) that configures the *Input Model, Data Configurations, Evaluation Criteria, Devices, Engine*, and *Search Strategy* modules. In *Input Model*, the operational domain of Hugging Face is defined, supporting the sentence-similarity task, while the MedSTS^8^ (Medical Sentence Similarity) (Wang Y. et al., 2018) Train and Test datasets serve as resources for model calibration through the *Data Configurations* module. *Evaluation Criteria* include accuracy, precision, recall, F1-score, and latency (average, maximum, minimum). The cache directories manage intermediate results, streamlining reproducibility and scalability. Optimization goals are defined algorithmically and adhered to strict parametric thresholds: a maximum performance degradation of 0.01% and a minimum latency improvement of 20%. In the *Device* module, local_system is designated as the GPU-supported system. *Engine and Search Strategy* employ the joint execution order with the TPE algorithm, for profiling and caching within the search space.

#### 4.1.3 ONNX runtime passes

Optimization begins with *OnnxConversion*, which converts PyTorch models to ONNX format (opset: 14) for hardware-agnostic execution. Subsequently, *OrtTransformersOptimization* module streamlines computational graphs by combining adjacent layers and pruning redundant nodes. *OrtMixedPrecision* enhances throughput and reduces memory usage by applying FP16^9^ arithmetic where applicable. Lastly, *OrtPerfTuning* profiles latency and throughput, performing runtime tuning^10^ in model configurations. The sequential application of these optimization steps enables modular result storage, allowing model assessment via Pareto frontier analysis.

#### 4.1.4 Search-optimized quantization

The INT8 (W8A8) quantization logic is implemented using SmoothQuant (Xiao et al., 2024), coordinating Intel Neural Compressor and IPEX (Intel Extension for PyTorch), together with Microsoft Olive and the ONNX Runtime backend. The *QOperator* format includes *QLinearMatMul, MatMulInteger, QLinearAdd*, and *QLinearRelu* operators, configured via custom JSON settings, in order to manage the transversal redistribution of quantization complexity through a smoothing factor α = 0.5, validated as optimal for the models from Microsoft Research and Cambridge LTL. The use of NGC containers streamlines the integration of the previous configuration script (user_script.py) and the calibration datasets, to ensure scalable model deployment on accelerated hardware, while retaining optimization objectives.

### 4.2 Main results and analysis

#### 4.2.1 DEFT 2020 evaluation campaign

Since, in our case study, there is no test dataset for inference matched with a training dataset for calibration, the MedSTS resources are used for this purpose, and inference is applied directly to this end as part of our approach. In addition, to quantify the efficiency of our optimization processes by means of performance, latency, and consumption metrics, we use the datasets from the two tasks of the DEFT 2020 Evaluation Campaign (Cardon et al., 2020), as they are broadly representative of our core objective of biomedical ontology alignment.^11^

In Task 1, which aims to identify the degree of semantic similarity between pairs of sentences, the input_cols parameter is set to [sentence1, sentence2], corresponding to the *source* and *target* fields, respectively. These are formatted as paired token sequences, and the label_cols parameter is set to [label] for the *mark* field, representing human-assigned scores from 0 to 5 indicating pairwise sentence-level semantic correspondence.

The same functional topology is transversally adapted for Task 2, concerning the identification of parallel sentences.^12^ In turn, the data from the latter are internally linked with the corresponding identifier present in the *num* field. This linkage linearly maps the inferential string yielding the highest cosine similarity score for each virtually tripartitioned segment, created based on the associated *id* of each data line. Thus, the correspondence with the identifier in [label], representing the *target* field, is ensured. The adoption of virtual compartment systems with three distinct conditions is introduced because the second task aims to identify, among three *target* sentences, the one that best corresponds to the *source* in terms of sentence-level parallelism.

#### 4.2.2 Configurational decorators

These configuration architectures are diligently designed using logging wrappers (decorators) to log the methodically engineered processing pipeline, and to generate the dataloader through HuggingfaceDataContainer. In practical application, this component enables robust evaluation metrics testing, thereby presenting a wide range of potential options.

#### 4.2.3 Task 1

The first task, focused on continuous semantic evaluation (Semantic Similarity Evaluation), presented complications in converting the models' inference outputs from cosine similarity percentages to the compliant evaluation format. Specifically, it has been found that, particularly for KRISSBERT (Zhang et al., 2022), the percentage scores of cosine semantic similarity are extremely high compared to the norm. This is presumably due to an improperly calibrated cross-entropy loss in the training of the cross-attention encoder, as cursorily reported in Microsoft Research's study, which results in the re-ranking score being maximized even for partial or incorrect entities. The model's inferences, while excelling in Named Entity Linking (NEL), lead to problems in cosine similarity score attribution. It is also advisable to review the linear layer applied to the encoding of the first [CLS] token to calculate the re-ranking score, as it has been proven that the score is very high even for nonsensical sentence pairs, potentially indicating poor discrimination. To address this, a feature scaling function using MinMaxScaler is manually added in the post_process_data module of HuggingfaceDataContainer, converging into a corrective fine-tuning. Its effectiveness is demonstrated in the following Table 1, which highlights evidence of errors from both Microsoft Research and Cambridge LTL.

**Table 1:** Examples highlighting a critical issue of score overestimation in the predictions made by the KRISSBERT and SapBERT-large models, which tend to disproportionately inflate the re-ranking scores, even for incomplete or incorrect entity matches.

**Source:** “*Royal jelly is a natural product very rich in vitamin B5 (C0001535), trace elements, acetylcholine (up to 0.1% by mass), and antibiotic factors notably active against Proteus and Escherichia coli B (C0001041), better known as colibacillus*.”
**Target:** “*Indeed, the smoke (C0037369) makes the bees (C0005108) perceive a fire, causing them to frantically gather honey reserves in their crop rather than defending their hive from the beekeeper*.”
KRISSBERT Prediction Score: 95%.
+ Corrective Fine-Tuning: 12%.
SapBERT-large Prediction Score: 43%.
+ Corrective Fine-Tuning: 7%.
**Source**: “*The degrees of originality (C0006267) and hybridization (C0020155) of these breeds, as well as their homogeneity, are poorly described*.”
**Target**: “*Without this precaution when opening a hive, the excitepment of a colony can rise, making it very dangerous (C0205166), given the number of bees (C0005108)*.”
KRISSBERT Prediction Score: 94%.
+ Corrective Fine-Tuning: 9%.
SapBERT-large Prediction Score: 37%.
+ Corrective Fine-Tuning: 5%.

This enabled the use of the official EDRM evaluation metric (Cardon et al., 2020), which measures the average relative distance to the solution as a micro-average. For each similarity value, the reference data *r*_*i*_ corresponds to the maximum possible distance between the system's predicted response and the data *d*_max_(*h*_*i*_, *r*_*i*_), formally defined in Equation 12:


(12) $$\begin{array}{l} \text{EDRM}=\frac{1}{n}\overset{n}{\underset{i=1}{\sum }}\left(1-\frac{d\left(h_{i},r_{i}\right)}{\text{dmax}\left(h_{i},r_{i}\right)}\right) \end{array}$$

**Figure 2:**
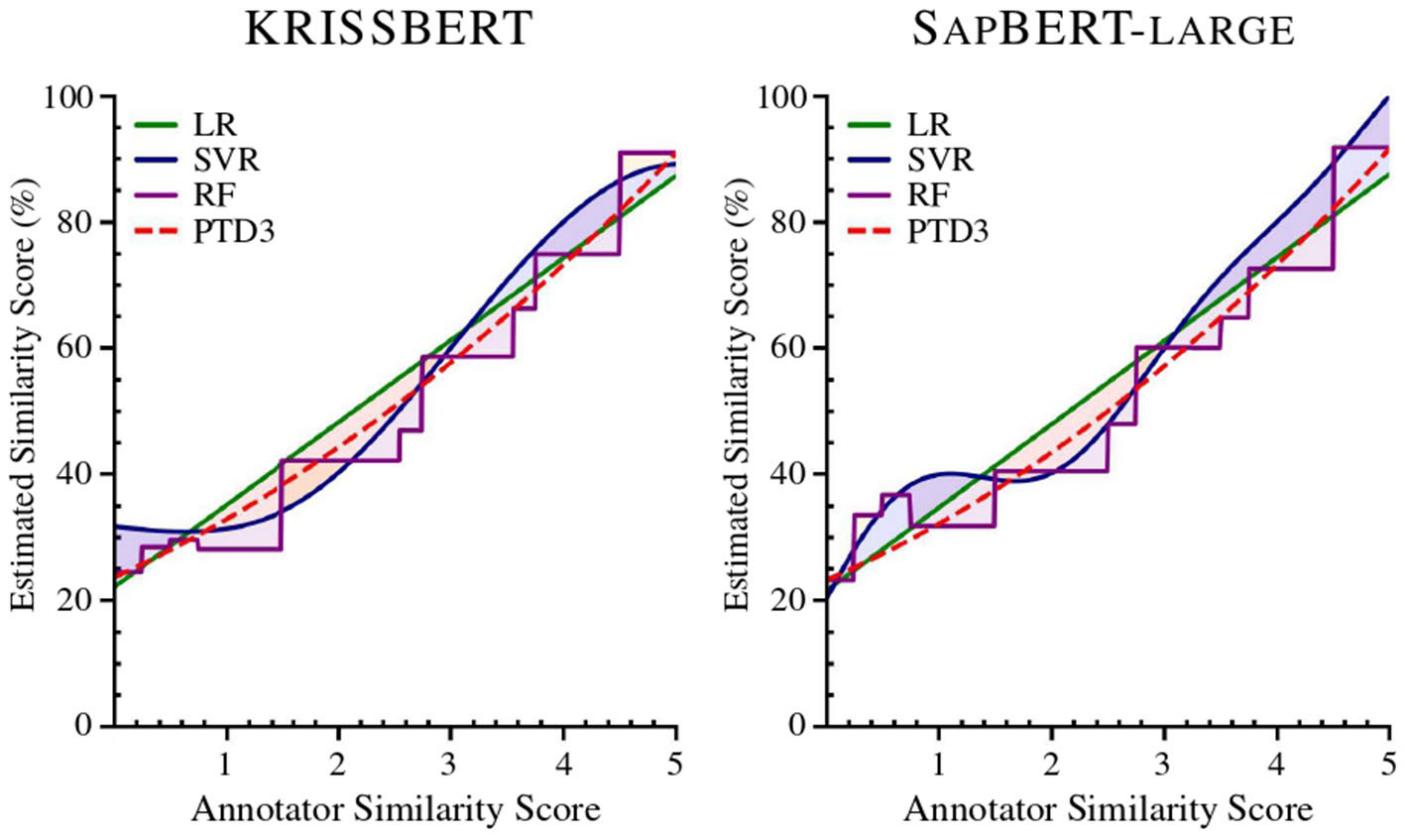
Regression comparison of the study models applied to Task 1, using Linear Regression (LR), Support Vector Regression (SVR), Random Forest Regression (RF), and a Polynomial Trendline with Degree 3 (PTD3). The Radial Basis Function (RBF) kernel is applied in the SVR model.

Our technique surpassed the previous FP32 state-of-the-art achieved by UASZ (Université Assane Seck de Ziguinchor) (Dramé et al., 2020), as presented in Table 2, and more statistically in Figure 2.

**Table 2:** Comparison of the study models, optimized to INT8 (W8A8) by Microsoft Olive, against the UASZ state-of-the-art (Dramé et al., 2020).

**Method**	**Task** @1		
	**EDRM**	**Spearman-correlation**	***p*** **-value**
KRISSBERT INT8	**0.8604**	0.8253	2.0724e-97
SapBERT-Large INT8	0.8593	**0.8289**	**2.5965e-99**
UASZ (Dramé et al., 2020), 1	0.7947	0.7528	4.3371e-76
UASZ (Dramé et al., 2020), 2	0.8217	0.7691	2.3769e-81
UASZ (Dramé et al., 2020), 3	0.7755	0.7769	5.5766e-84

The metrics include EDRM, Spearman's rank correlation, and *p*-values. Bold values indicate the highest performance per column.

#### 4.2.4 Task 2

In the second task of DEFT 2020, which closely aligns with the conditions of our main mission, the evaluation metric consists of a classification-based assessment: the Mean Average Precision (MAP), formulated in Equation 13, is computed as the mean of the non-interpolated precisions $P\left(I_{i}^{j}\right)$ at each position in the ranked list of hypotheses, for each of the *n*_*i*_ correct answers $I_{i}^{j}$ associated with a given *source* sentence *S*_*i*_:


(13) $$\begin{array}{l} \text{MAP}=\frac{1}{N}\overset{N}{\underset{i=1}{\sum }}\frac{1}{n_{i}}\overset{n_{i}}{\underset{j=1}{\sum }}P\left(I_{i}^{j}\right) \end{array}$$

As detailed in Table 3, our approach has significantly outperformed the previous ones from both the University of Sorbonne (Buscaldi et al., 2020) and Synapse (Teissèdre et al., 2020).

**Table 3:** Comparison of the study models, optimized to INT8 (W8A8) by Microsoft Olive, against the state-of-the-art benchmarks from Sorbonne (Buscaldi et al., 2020) and Synapse (Teissèdre et al., 2020).

**Method**	**Task** @2			
	**MAP-1**	**MAP-2**	**MAP-3**	**Mean**
KRISSBERT INT8	0.9977	**0.9991**	**1**	0.9989
SapBERT-Large INT8	**1**	0.9974	**1**	**0.9991**
Sorbonne (Buscaldi et al., 2020)	0.9887	0.9887	0.9887	0.9887
Synapse (Teissèdre et al., 2020)	0.9906	0.9849	0.9396	0.9717

The metrics include MAP classification scores (MAP-1, MAP-2, MAP-3), with their respective mean values used as the evaluation standard. Bold values indicate the highest performance per column.

#### 4.2.5 The impact of search-optimized quantization

Trade-off metrics between performance,^13^ latency, power consumption, and estimated carbon emissions^14^ are rigorously quantified using the huggingface_metrics backend, as reported in Table 4.

**Table 4:** Comparison of performance, latency, and consumption metrics for KRISSBERT and SapBERT-large models before and after optimization across the two tasks of the DEFT 2020 Evaluation Campaign.

	**Performance**				**Latency**			**Consumption**		
**Task** @1	**Accuracy**	**Precision**	**Recall**	**F1-score**	**Latency-avg**	**Latency-max**	**Latency-min**	**Size**	**GPU energy**	**CO2**
KRISSBERT (Zhang et al., 2022)	0.8886	0.9047	0.8920	0.8983	19.9143	20.2043	19.6533	438	2.2127	1.0510
+ Microsoft Olive	0.8886	0.9047	0.8920	0.8983	1.2114	1.2165	1.2051	166.44	0.1346	0.0639
SapBERT-large (Liu et al., 2021)	0.8808	0.8851	0.8937	0.8894	64.0251	64.3159	63.7649	2293.76	7.1139	3.3791
+ Microsoft Olive	0.8808	0.8851	0.8937	0.8894	3.0494	3.0562	3.0453	756.94	0.3388	0.1609
**Task** @2	**MAP-1**	**MAP-2**	**MAP-3**	**Mean**	**Latency-avg**	**Latency-max**	**Latency-min**	**Size**	**GPU energy**	**CO2**
KRISSBERT (Zhang et al., 2022)	0.9977	0.9991	1	0.9989	55.3579	55.6289	55.1095	438	6.1509	2.9217
+ Microsoft Olive	0.9977	0.9991	1	0.9989	3.0276	3.0351	3.0228	171.58	0.3364	0.1598
SapBERT-large (Liu et al., 2021)	1	0.9974	1	0.9991	185.5632	185.8308	185.3122	2293.76	20.6181	9.7936
+ Microsoft Olive	1	0.9974	1	0.9991	9.7195	9.7255	9.7138	762.13	1.0799	0.5130

Blue indicates maintained performance metrics in both the original and the algorithm-driven optimized models, while the transition to Green indicates improvements in both timing and resource utilization. In both cases, the optimization process yields reduced latency and energy consumption, while preserving overall performance. All results refer to inference.

**Figure 3:**
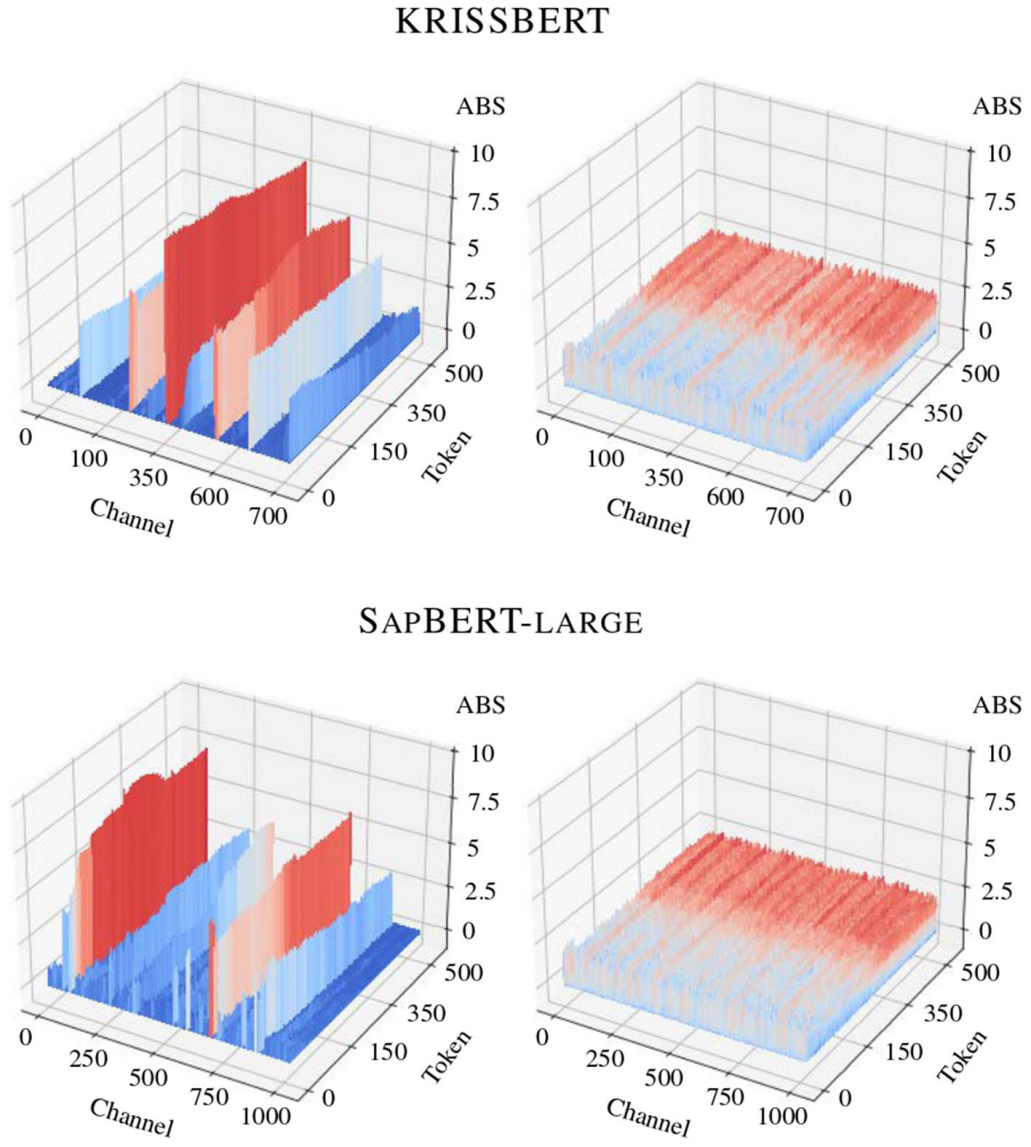
Impact of search-optimized quantization on the distribution of activations in the models under study, before and after optimization. Several channels in the original activation map display significantly high magnitudes, while the variance within a particular activation channel is consistently and notably low throughout.

For observational purposes, the effectiveness of the process is validated during the verification phase using the *Quantization Debug* module of ONNX Runtime, which provides a detailed graphical representation of the redistribution of computational complexity.^15^ For simplicity, the comparison between the activation tensors from the original computation graph and its quantized counterpart is demonstrated in Figure 3.

#### 4.2.6 Biomedical ontology alignment

**Figure 4:**
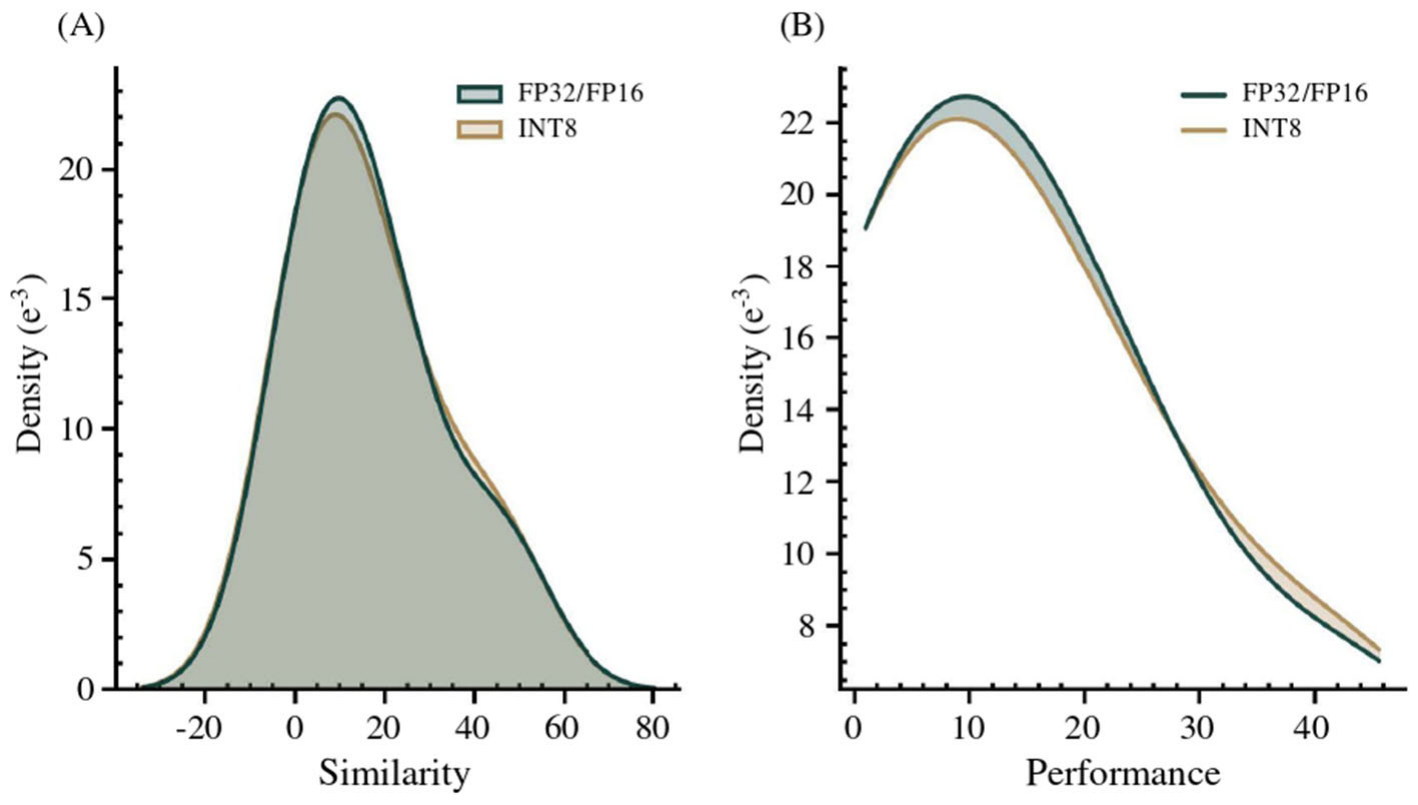
**(A)** Gaussian kernel density estimation of performance scores across floating-point (FP32/FP16) and quantized (INT8) model formats; **(B)** Detailed view of distribution shifts induced by format variation.

Upon completion of the vocabulary, aligned using the np.argmax matrix logic (Section 3.2) between the LEX and MRCONSO domains, a manual verification is conducted using the six-point rating scale. The resulting alignments, obtained from two quantized transformer models, are then merged using the complementarity-based aggregation strategy, which iteratively integrates non-overlapping alignments in descending order of rating to increase coverage while preserving precision. The comparative rating distribution is reported in Table 5, followed by a Gaussian analysis in Figure 4, which illustrates overall performance consistency across model formats.

**Table 5:** Comparison of manual rating distributions over scores @*k* for vocabulary alignments across individual models and their complementary combination.

**Model**	**@0**	**@1**	**@2**	**@3**	**@4**	**@5**
KRISSBERT INT8	186	798	1,343	3,028	4,098	7,941
SapBERT-Large INT8	205	687	1,403	2,928	4,169	8,002
+ Complementarity	/	/	/	897	5,473	11,024

## 5 Conclusion

We present a cutting-edge, optimization-driven solution for biomedical ontology alignment, leveraging Microsoft Olive, ONNX Runtime, and a novel quantization strategy implemented through Intel Neural Compressor and IPEX. Empirical evaluations demonstrate an average 20 × inference speed-up and a 70% reduction in memory usage, achieved without compromising performance. Validated across multiple datasets, our approach establishes new state-of-the-art results in all evaluated domains.

Beyond reducing deployment costs, our approach enables scalability across resource-limited settings. By providing a robust, turnkey framework that preserves accuracy while maximizing efficiency, we contribute to the broader democratization of deep learning technologies. Future work will explore the application of this methodology to other domains, potentially extending its benefits across a wide range of research areas.

## 6 Limitations

The performance of our methods is influenced by external factors, including hardware configurations, software dependencies, and environmental conditions. A thorough analysis of these elements and their impact is essential for practical deployment and real-world applications. Such analysis should also be extended to different model architectures, including large language models.

## Code availability

The code required to reproduce the findings is available at the GitHub repository https://github.com/OussamaBouaggad/Quantization and is distributed under the MIT License.

## Data Availability

UMLS (Bodenreider, 2004) is licensed to individuals for research purposes. CNRS resources are provided under the End User License Agreement (EULA), as are the DEFT 2020 Evaluation Campaign datasets (Cardon et al., 2020). The MedSTS dataset (Wang Y. et al., 2018) is freely available for public use. KRISSBERT (Zhang et al., 2022) and SapBERT-large (Liu et al., 2021) models are distributed under the MIT License, as are Microsoft Olive and ONNX Runtime. ScispaCy (Neumann et al., 2019), Intel Neural Compressor, and IPEX (Intel Extension for PyTorch) are released under the Apache License 2.0.

## Footnotes

^1^The official UMLS resource is accessible at https://www.nlm.nih.gov/research/umls/index.html.

^2^Entities include classes, instances, properties, relationships, data types, annotations, and cardinality constraints.

^3^A class of an ontology typically contains a list of labels (via annotation properties such as *rdfs:label*) that serve as alternative class names, descriptions, synonyms, or aliases.

^4^The search spaces of all passes are combined and jointly evaluated to find optimal parameters, using Optuna's TPESampler.

^5^Such an outcome involves Mul operations without folding, optimized in IPEX through system-level automatic fusion.

^6^Such behavior is particularly noticeable in scenarios involving activation outliers, where standard quantization methods struggle to maintain consistency across input distributions.

^7^The concatenation of diverse and evolving domains ensures comprehensive biomedical alignment (Koptient and Grabar, 2020).

^8^MedSTS, which incorporates UMLS concepts, is designed to measure biomedical semantic textual similarity, including sentence pairs annotated with similarity scores.

^9^In the present configuration, Float16 precision is enabled for CUDAExecutionProvider but disabled for TensorRTExecutionProvider, balancing compatibility and computational gains.

^10^The proposed runtime tuning enhances model calibration and inference through dynamic architectural optimization.

^11^In the Train module, the pretrained models are calibrated by framing optimal model optimizations aligned with the highest hardware performance capabilities, whereas in the Test module, the evaluation metrics are established.

^12^The parallelism of the sentences is related to the simple-complex relationship, ergo one of the simple sentences (*target*) is always derived from the complex sentence (*source*).

^13^In Task 1, given the specificity of the EDRM evaluation, the accuracy, precision, recall, and F1-Score metrics are applied instead.

^14^The carbon emissions are calculated based on the GPU emission factor of 0.475 kg CO_2_ per kWh.

^15^The module handles activation outliers, which commonly fall within the absolute value range of 2.5 to 5, with extreme cases peaking above 7.5, thus affecting scaling factors.

## References

[B1] Balaskas K. Karatzas A. Sad C. Siozios K. Anagnostopoulos I. Zervakis G.. (2024). Hardware-aware DNN compression via diverse pruning and mixed-precision quantization. IEEE Trans. Emerg. Top. Comput. 12, 1079–1092. 10.1109/TETC.2023.3346944

[B2] Bodenreider O. (2004). The unified medical language system (UMLS): integrating biomedical terminology. Nucleic Acids Res. 32, D267-D270. 10.1093/nar/gkh061 14681409 PMC308795

[B3] Buscaldi D. Felhi G. Ghoul D. Le Roux J. Lejeune G. Zhang X. (2020). “Calcul de similarité entre phrases : quelles mesures et quels descripteurs? (sentence similarity: a study on similarity metrics with words and character strings),” in Actes de la 6e conférence conjointe Journées d'Études sur la Parole (JEP, 33e édition), Traitement Automatique des Langues Naturelles (TALN, 27e édition), Rencontre des Étudiants Chercheurs en Informatique pour le Traitement Automatique des Langues (RÉCITAL, 22e édition) Atelier DÉfi Fouille de Textes, eds. R. Cardon, N. Grabar, C. Grouin, T. Hamon (Nancy, France: ATALA et AFCP), 14–25.

[B4] Cardon R. Grabar N. Grouin C. Hamon T. (2020). “Présentation de la campagne d'évaluation DEFT 2020 : similarité textuelle en domaine ouvert et extraction d'information précise dans des cas cliniques (Presentation of the DEFT 2020 challenge: Open domain textual similarity and precise information extraction from clinical cases),” in Actes de la 6e conférence conjointe Journées d'Études sur la Parole (JEP, 33e édition), Traitement Automatique des Langues Naturelles (TALN, 27e édition), Rencontre des Étudiants Chercheurs en Informatique pour le Traitement Automatique des Langues (RÉCITAL, 22e édition). Atelier DÉfi Fouille de Textes, eds. R. Cardon, N. Grabar, C. Grouin, T. Hamon (Nancy, France: ATALA et AFCP), 1–13.

[B5] Carreira-Perpiñán M. (2017). Model compression as constrained optimization, with application to neural nets. Part I: General framework. arXiv [Preprint]. arXiv:1707.01209. 10.48550/arXiv.1707.01209

[B6] Chen J. Jiménez-Ruiz E. Horrocks I. Antonyrajah D. Hadian A. Lee J. (2021). “Augmenting ontology alignment by semantic embedding and distant supervision,” in The Semantic Web, eds. R. Verborgh, K. Hose, H. Paulheim, P.-A. Champin, M. Maleshkova, O. Corcho, et al. (Cham: Springer International Publishing), 392–408. 10.1007/978-3-030-77385-4_23

[B7] Courbariaux M. Bengio Y. David J.-P. (2015). “Binaryconnect: training deep neural networks with binary weights during propagations,” in Proceedings of the 28th International Conference on Neural Information Processing Systems - Volume 2, NIPS'15 (Cambridge, MA, USA: MIT Press), 3123–3131.

[B8] Dagan I. Dolan B. Magnini B. Roth D. (2009). Recognizing textual entailment: rational, evaluation and approaches. Nat. Lang. Eng. 15, i–xvii. 10.1017/S1351324909990209

[B9] Dramé K. Sambe G. Diop I. Faty L. (2020). “Approche supervisée de calcul de similarité sémantique entre paires de phrases (supervised approach to compute semantic similarity between sentence pairs),” in Actes de la 6e conférence conjointe Journées d'Études sur la Parole (JEP, 33e édition), Traitement Automatique des Langues Naturelles (TALN, 27e édition), Rencontre des Étudiants Chercheurs en Informatique pour le Traitement Automatique des Langues (RÉCITAL, 22e édition). Atelier DÉfi Fouille de Textes, eds. R. Cardon, N. Grabar, C. Grouin, T. Hamon (Nancy, France: ATALA et AFCP), 49–54.

[B10] Euzenat J. Shvaiko P. (2007). Ontology Matching. Springer: New York.

[B11] Fang L. Chen Q. Wei C.-H. Lu Z. Wang K. (2023). Bioformer: An efficient transformer language model for biomedical text mining. arXiv preprint arXiv:2302.01588. 10.48550/arXiv.2302.0158836945685

[B12] Faria D. Pesquita C. Santos E. Palmonari M. Cruz I. F. Couto F. M. (2013). “The agreementmakerlight ontology matching system,” in On the Move to Meaningful Internet Systems: OTM 2013 Conferences, eds. R. Meersman, H. Panetto, T. Dillon, J. Eder, Z. Bellahsene, N. Ritter et al. (Berlin, Heidelberg: Springer Berlin Heidelberg), 527–541. 10.1007/978-3-642-41030-7_38

[B13] Frankle J. Carbin M. (2019). “The lottery ticket hypothesis: finding sparse, trainable neural networks,” in ICLR (OpenReview.net). Available online at: http://dblp.uni-trier.de/db/conf/iclr/iclr2019.html#FrankleC19

[B14] Gu Y. Tinn R. Cheng H. Lucas M. Usuyama N. Liu X.. (2021). Domain-specific language model pretraining for biomedical natural language processing. ACM Trans. Comput. Healthcare 3, 1–23. 10.1145/3458754

[B15] Guo Y. Yao A. Chen Y. (2016). Dynamic network surgery for efficient DNNs. arXiv preprint arXiv:1608.04493. 10.48550/arXiv.1608.04493

[B16] Han S. Mao H. Dally W. J. (2015). Deep compression: Compressing deep neural network with pruning, trained quantization and huffman coding. arXiv preprint arXiv:1510.00149. 10.48550/arXiv.1510.00149

[B17] Hassibi B. Stork D. (1992). “Second order derivatives for network pruning: optimal brain surgeon,” in Proceedings of the 6th International Conference on Neural Information Processing Systems, Denver, CO, NIPS'92 (San Francisco, CA: Morgan-Kaufmann), 164–171.

[B18] He Y. Chen J. Antonyrajah D. Horrocks I. (2021). “Biomedical ontology alignment with BERT,” in Proceedings of the 16th International Workshop on Ontology Matching co-located with the 20th International Semantic Web Conference (ISWC 2021), CEUR Workshop Proceedings, vol. 3063, eds. P. Shvaiko, J. Euzenat, E. Jiménez-Ruiz, O. Hassanzadeh, and C. Trojahn (CEUR-WS.org), 1–12. Available online at: https://ceur-ws.org/Vol-3063/om2021_LTpaper1.pdf

[B19] Hinton G. Vinyals O. Dean J. (2015). Distilling the knowledge in a neural network. arXiv preprint arXiv:1503.02531. 10.48550/arXiv.1503.02531

[B20] Huai S. Kong H. Luo X. Liu D. Subramaniam R. Makaya C.. (2023). On hardware-aware design and optimization of edge intelligence. IEEE Des. Test 40, 149–162. 10.1109/MDAT.2023.3307558

[B21] Jacob B. Kligys S. Chen B. Zhu M. Tang M. Howard A.. (2018). “Quantization and training of neural networks for efficient integer-arithmetic-only inference,” in Proceedings of the IEEE Conference on Computer Vision and Pattern Recognition (CVPR), 2704–2713. 10.1109/CVPR.2018.00286

[B22] Ji Z. Wei Q. Xu H. (2020). BERT-based ranking for biomedical entity normalization. AMIA Summits Transl. Sci. Proc. 2020:269. Available online at: https://arxiv.org/pdf/1908.03548.pdf 32477646 PMC7233044

[B23] Jiménez-Ruiz E. Cuenca Grau B. (2011). “LogMap: logic-based and scalable ontology matching,” in The Semantic Web-ISWC 2011, eds. L. Aroyo, C. Welty, H. Alani, J. Taylor, A. Bernstein, L. Kagal, et al. (Berlin, Heidelberg: Springer Berlin Heidelberg), 273–288. 10.1007/978-3-642-25073-6_18

[B24] Kim S. Gholami A. Yao Z. Mahoney M. W. Keutzer K. (2021). I-BERT: integer-only BERT quantization. arXiv preprint arXiv:2101.01321. 10.48550/arXiv.2101.01321

[B25] Kolyvakis P. Kalousis A. Kiritsis D. (2018). “DeepAlignment: unsupervised ontology matching with refined word vectors,” in Proceedings of the 2018 Conference of the North American Chapter of the Association for Computational Linguistics: Human Language Technologies, Volume 1 (Long Papers), eds. M. Walker, H. Ji, A. Stent (New Orleans, Louisiana: Association for Computational Linguistics), 787–798. 10.18653/v1/N18-1072

[B26] Koptient A. Grabar N. (2020). “Rated lexicon for the simplification of medical texts,” in The Fifth International Conference on Informatics and Assistive Technologies for Health-Care, Medical Support and Wellbeing HEALTHINFO 2020, Porto, Portugal. 10.3233/SHTI210170

[B27] Lambrix P. (2004). “Ontologies in bioinformatics and systems biology,” in Artificial Intelligence Methods And Tools For Systems Biology, eds. D. Werner, and A. Francisco (Dordrecht: Springer Netherlands), 129–145. 10.1007/1-4020-2865-2_8

[B28] LeCun Y. Denker J. Solla S. (1989). “Optimal brain damage,” in Proceedings of the 3rd International Conference on Neural Information Processing Systems, NIPS'89 (Cambridge, MA: MIT Press), 598–605.

[B29] Lee J. Yoon W. Kim S. Kim D. Kim S. So C. H.. (2020). BioBERT: a pre-trained biomedical language representation model for biomedical text mining. Bioinformatics 36, 1234–1240. 10.1093/bioinformatics/btz682 31501885 PMC7703786

[B30] Liu F. Shareghi E. Meng Z. Basaldella M. Collier N. (2021). Self-alignment pretraining for biomedical entity representations. in Proceedings of the 2021 Conference of the North American Chapter of the Association for Computational Linguistics: Human Language Technologies, eds. K. Toutanova, A. Rumshisky, L. Zettlemoyer, D. Hakkani-Tur, I. Beltagy, S. Bethard, et al. (Association for Computational Linguistics), 4228–4238. 10.18653/v1/2021.naacl-main.334

[B31] Logeswaran L. Chang M.-W. Lee K. Toutanova K. Devlin J. Lee H. (2019). “Zero-shot entity linking by reading entity descriptions,” in Proceedings of the 57th Annual Meeting of the Association for Computational Linguistics, eds. A. Korhonen, D. Traum, L. Màrquez (Florence, Italy: Association for Computational Linguistics), 3449–3460. 10.18653/v1/P19-1335

[B32] Micikevicius P. Narang S. Alben J. Diamos G. Elsen E. Garcia D.. (2018). “Mixed precision training,” in International Conference on Learning Representations. Available online at: https://openreview.net/forum?id=r1gs9JgRZ

[B33] Mikolov T. Chen K. Corrado G. Dean J. (2013). “Efficient estimation of word representations in vector space,” in 1st International Conference on Learning Representations, ICLR 2013, Scottsdale, Arizona, USA, May 2-4, 2013, Workshop Track Proceedings. Available online at: http://arxiv.org/abs/1301.3781

[B34] Nagel M. van Baalen M. Blankevoort T. Welling M. (2019). Data-free quantization through weight equalization and bias correction. arXiv preprint arXiv:1906.04721. 10.48550/arXiv.1906.04721

[B35] Neumann M. King D. Beltagy I. Ammar W. (2019). “ScispaCy: fast and robust models for biomedical natural language processing,” in Proceedings of the 18th BioNLP Workshop and Shared Task, eds. D. Demner-Fushman, K. B. Cohen, S. Ananiadou, J. Tsujii (Florence, Italy: Association for Computational Linguistics), 319–327. 10.18653/v1/W19-5034

[B36] Nowlan S. J. Hinton G. E. (1992). Simplifying neural networks by soft weight-sharing. Neural Comput. 4, 473–493. 10.1162/neco.1992.4.4.473

[B37] Park J.-H. Kim K.-M. Lee S. (2022). Quantized sparse training: a unified trainable framework for joint pruning and quantization in DNNs. ACM Trans. Embed. Comput. Syst. 21:60. 10.1145/3524066

[B38] Qu Z. Zhou Z. Cheng Y. Thiele L. (2020). “Adaptive loss-aware quantization for multi-bit networks,” in Proceedings of the IEEE/CVF Conference on Computer Vision and Pattern Recognition (CVPR) (IEEE), 7985–7994. 10.1109/CVPR42600.2020.00801

[B39] Rakka M. Fouda M. E. Khargonekar P. Kurdahi F. (2022). Mixed-precision neural *networks*: a survey. arXiv preprint arXiv:2208.06064. 10.48550/arXiv.2208.06064 38683716

[B40] Rokh B. Azarpeyvand A. Khanteymoori A. (2023). A comprehensive survey on model quantization for deep neural networks in image classification. ACM Trans. Intell. Syst. Technol. 14:97. 10.1145/3623402

[B41] Roy S. Mehera R. Pal R. Bandyopadhyay S. (2023). Hyperparameter optimization for deep neural network models: a comprehensive study on methods and techniques. Innov. Syst. Softw. Eng. 21, 1–12. 10.1007/s11334-023-00540-3

[B42] Schaefer C. J. Lambert-Shirzad N. Zhang X. Chou C. Jablin T. Li J.. (2023). Augmenting Hessians with inter-layer dependencies for mixed-precision post-training quantization. arXiv preprint arXiv:2306.04879. 10.48550/arXiv.2306.04879

[B43] Shen S. Zhen D. Ye J. Ma L. Yao Z. Gholami A.. (2020). Q-BERT: hessian based ultra low precision quantization of BERT. Proc. AAAI Conf. Artif. Intell. 34, 8815–8821. 10.1609/aaai.v34i05.6409

[B44] Shivapakash S. Jain H. Hellwich O. Gerfers F. (2020). “A power efficient multi-bit accelerator for memory prohibitive deep neural networks,” in 2020 IEEE International Symposium on Circuits and Systems (ISCAS), 1–5. 10.1109/ISCAS45731.2020.9180868

[B45] Sung M. Jeon H. Lee J. Kang J. (2020). “Biomedical entity representations with synonym marginalization,” in Proceedings of the 58th Annual Meeting of the Association for Computational Linguistics (ACL) (Association for Computational Linguistics), 3641–3650. 10.18653/v1/2020.acl-main.335

[B46] Teissèdre C. Belkacem T. Arens M. (2020). “Similarité sémantique entre phrases : apprentissage par transfert interlingue (semantic sentence similarity: multilingual transfer learning),” in Actes de la 6e conférence conjointe Journées d'Études sur la Parole (JEP, 33e édition), Traitement Automatique des Langues Naturelles (TALN, 27e édition), Rencontre des Étudiants Chercheurs en Informatique pour le Traitement Automatique des Langues (RÉCITAL, 22e édition), eds. R. Cardon, N. Grabar, C. Grouin, T. Hamon (Atelier DÉfi Fouille de Textes: Nancy, France. ATALA et AFCP), 97–107.

[B47] Vaswani A. Shazeer N. Parmar N. Uszkoreit J. Jones L. Gomez A. N.. (2017). “Attention is all you need,” in Proceedings of the 31st International Conference on Neural Information Processing Systems, Long Beach, CA, NIPS'17 (Red Hook, NY: Curran Associates, Inc.), 6000–6010.

[B48] Wang L. L. Bhagavatula C. Neumann M. Lo K. Wilhelm C. Ammar W. (2018). “Ontology alignment in the biomedical domain using entity definitions and context,” in Proceedings of the BioNLP 2018 workshop, eds. D. Demner-Fushman, K. B. Cohen, S. Ananiadou, J. Tsujii (Melbourne, Australia: Association for Computational Linguistics), 47–55. 10.18653/v1/W18-2306

[B49] Wang Y. Afzal N. Fu S. Wang L. Shen F. Rastegar-Mojarad M.. (2018). MedSTS: A resource for clinical semantic textual similarity. arXiv preprint arXiv:1808.09397. 10.48550/arXiv.1808.09397

[B50] Wang Y. Lu Y. Blankevoort T. (2020). “Differentiable joint pruning and quantization for hardware efficiency,” in Computer Vision - ECCV 2020: 16th European Conference, Glasgow, UK, August 23-28, 2020, Proceedings, Part XXIX (Berlin, Heidelberg: Springer-Verlag), 259–277. 10.1007/978-3-030-58526-6_16

[B51] Wu H. Judd P. Zhang X. Isaev M. Micikevicius P. (2020). Integer quantization for deep learning inference: principles and empirical evaluation. arXiv preprint arXiv:2004.09602. 10.48550/arXiv.2004.09602

[B52] Wu L. Petroni F. Josifoski M. Riedel S. Zettlemoyer L. (2020). “Scalable zero-shot entity linking with dense entity retrieval,” in Proceedings of the 2020 Conference on Empirical Methods in Natural Language Processing (EMNLP), eds. B. Webber, T. Cohn, Y. He, Y. Liu (Association for Computational Linguistics), 6397–6407. 10.18653/v1/2020.emnlp-main.519

[B53] Xiao G. Lin J. Seznec M. Wu H. Demouth J. Han S. (2024). SmoothQuant: Accurate and efficient post-training quantization for large language models. arXiv preprint arXiv:2211.10438. 10.48550/arXiv.2211.10438

[B54] Xu D. Zhang Z. Bethard S. (2020). “A generate-and-rank framework with semantic type regularization for biomedical concept normalization,” in Proceedings of the 58th Annual Meeting of the Association for Computational Linguistics, eds. J. Dan, C. Joyce, S. Natalie, and T. Joel (Association for Computational Linguistics), 8452–8464. 10.18653/v1/2020.acl-main.748

[B55] Xu Z. Hsu Y.-C. Huang J. (2017). Training shallow and thin networks for acceleration via knowledge distillation with conditional adversarial networks. arXiv preprint arXiv:1709.00513. 10.48550/arXiv.1709.00513

[B56] Yang H. Gui S. Zhu Y. Liu J. (2020). “Automatic neural network compression by sparsity-quantization joint learning: a constrained optimization-based approach,” in 2020 IEEE/CVF Conference on Computer Vision and Pattern Recognition (CVPR) (Los Alamitos, CA: IEEE Computer Society), 2175–2185. 10.1109/CVPR42600.2020.00225

[B57] Yu P.-H. Wu S.-S. Klopp J. P. Chen L.-G. Chien S.-Y. (2020). Joint pruning and quantization for extremely sparse neural networks. arXiv preprint arXiv:2010.01892. 10.48550/arXiv.2010.01892

[B58] Zhang S. Cheng H. Vashishth S. Wong C. Xiao J. Liu X.. (2022). “Knowledge-rich self-supervision for biomedical entity linking,” Findings of the Association for Computational Linguistics: EMNLP 2022, eds. Y. Goldberg, Z. Kozareva, Y. Zhang (Abu Dhabi: Association for Computational Linguistics), 868–880. 10.18653/v1/2022.findings-emnlp.61

[B59] Zhao R. Hu Y. Dotzel J. Sa C. D. Zhang Z. (2019). Improving neural network quantization without retraining using outlier channel splitting. arXiv preprint arXiv:1901.09504. 10.48550/arXiv.1901.09504

