# Supplementary material for: Search-optimized quantization in biomedical ontology alignment
Source: Front Artif Intell. 2025 Oct 10;8:1662984. doi: 10.3389/frai.2025.1662984 (PMC12551506; doi:10.3389/frai.2025.1662984)
Supplement: Supplementary file 1 [file Data_Sheet_1.pdf]

Supplementary Material

1 FINE-TUNING ARCHITECTURE

The fine-tuning configuration of the respective models involved defining architecturally optimal setups to ensure stability and effectiveness in tasks 1 and 2 of the DEFT 2020 Evaluation Campaign. The data preparation for the training modules associated with them adhered to the methodology reported for biomedical alignment within the main scope, unifying task initialization into a cohesive and standardized approach. In the Microsoft Research model, the Adam optimizer, in its ADAMW variant, is employed with an initial learning rate of 1×10^{-5} and a learning rate scheduler, `ReduceLROnPlateau`, which reduces the learning rate by a factor of 0.1 if performance on the validation set does not improve over three consecutive epochs. The framework utilized is the HUGGINGFACE TRAINER, which streamlines the integration of model configuration, dataset preprocessing, evaluation metrics, and resource management within a unified execution pipeline. The `batch_size` is set to 8, with a dropout rate of 0.1 applied to mitigate overfitting. Additionally, the `pmask` and `preplace` functions are implemented during tokenization with a probability of 0.2, thereby introducing controlled variability into the input data. The temperatures τ and π are consistently maintained at 1.0 to ensure stable gradient flow. In the Cambridge LTL model, similarly to the Microsoft Research setup, the ADAMW optimizer is applied with a learning rate of 1×10^{-5}, but with a weight decay rate of 1×10^{-2}. Although automatic mixed precision is originally preferred by researchers at Cambridge LTL, it is disabled in favor of maximum precision. The preprocessed and encoded data are partitioned into batches of 8 samples, across 3 epochs, with training likewise performed using the HUGGINGFACE TRAINER. Fine-tuning for both models is conducted on NVIDIA A100 GPUs, with all parameters carefully configured with respect to the nuanced connotative context specific to each of the two distinct and interrelated tasks.

In Task 1, the models undergo multi-class fine-tuning, utilizing a customized grading scale tailored to the scalably distorted cosine similarity outputs of the study models. This process involved converting the original labels from the *t1-train* module into a percentage format of cosine similarity, scaled in accordance with the range of outputs obtained during an initial inference on *t1-test*. This manipulation is necessary to properly test this technique statistically, allowing it to capture more nuances in the pairs of sentences of interest (*source* and *target*) compared to traditional binary class fine-tuning. The loss function is adapted using a combined loss integrating categorical cross-entropy and mean squared error, also known as MSE. This choice is motivated by the fact that categorical cross-entropy loss is suitable for multi-class classification and allows the model to learn to correctly distinguish between different classes of semantic similarity. By incorporating mean squared error, predictions that deviate substantially from the actual similarity values are penalized, thus improving the model's accuracy in recognizing semantic gradation and its performance on the official evaluation metrics. The weights of the losses, α and β, are balanced at 0.5, ensuring harmonious optimization, in accordance with Equation S1:

$$\alpha \times \text{Categorical Cross-Entropy} + \beta \times \text{MSE} \tag{S1}$$

Within this analytical framework, it is important to note that, prior to the optimization process, both modules (Train and Test) undergo a binary balancing between the positive and negative classes, the latter being slightly predominant. This is achieved through an automated undersampling method selectively applied to correct errors arising from discrepancies in human evaluation, notably when there is a significant

distance between the *mark* and *mean* fields. An illustrative case is provided by the pair with identifier *id* 413 in the *t1-train* module, where the *mark* field has a value of 5, projectively corresponding to a positive label, yet the *mean* field holds a projectively negative value of 2.1. The related *scores* field is $[3, 0.5, 2, 5, 0]$, which logically should not yield a *mark* value of 5, revealing an evaluation coherence error. Or, in the *t1-test* module, by the pair with identifier *id* 38, where there is a projectively positive value of 4 in the *mark* field associated with the lower value of 2 in the *mean* field, with the observed values $[2, 1, 0, 3, 4]$ in the *scores* field. This corrective adjustment did not significantly affect the original data composition, as both the distributions examined in each module remained quantitatively similar. Nevertheless, it contributed to more reliable and representative evaluation criteria, mitigating instability introduced by inconsistent assessments.

In Task 2, the models are trained with the aim of improving the identification of correspondences between pairs of sentences of interest (*source* and *target*) through the calculation of cosine similarity. This objective is pursued by adhering to the underlying logic of simple-complex relationships in sentence parallelism, taking into account three distinct conditions within each compartment. This compartmental structure is aligned with the purpose of the task, namely to evaluate three candidate *target* sentences and determine the one that exhibits the highest degree of parallelism with the *source* field. Given that a response is always expected from the three provided *target* sentences, the task requires the identification of a suitable parallel sentence for each corresponding set of *source* and *target* sentences. In reconsideration, the concept of sentence parallelism is rooted in the simple-complex relationship, wherein the *source* sentence represents complex content, while the simple sentences convey simplified or less complex content, resulting from derivation. A list of positive pairs (*source* and *target*), sequentially initialized with `[CLS]` tokens and then concatenated and delimited with `[SEP]` separator tokens, is generated by combining the respective correspondences with *target*. This sequence is then passed through a higher classification layer, which identifies the correct alignment via the correspondence with *num* in each tripartite compartment for all unique identifiers *id*. In this context, the loss function is simplified in comparison to the previous one, as it is based on the cross-entropy loss. This allows the models to learn to accurately minimize the loss by directly comparing the predictions with the true labels (*target*). These parametric finalizations involve prototyping a series of trial calibrations in the respective Test phases, thereby determining the optimal values according to the functional properties of each model.

2 WHY IS IT IMPORTANT TO APPLY THE SENTENCE-SIMILARITY MODALITY?

Upon in-depth consideration, opting for a conventional `text-classification` task would have resulted in an evaluation metric not suitable for our study, as the six-point similarity scale employed by the five expert annotators of the DEFT 2020 Evaluation Campaign is explicitly designed to assess contextual cosine semantic similarity. Therefore, quantifying each sample using cosine similarity and subsequently adapting the inference output distribution to match the official multi-label evaluation format proves to be the most appropriate approach. For methodological purposes, the task is framed in `sentence-similarity` mode to demonstrate the benefits of optimization, specifically maintaining performance metrics while simultaneously reducing latency and resource consumption. This is carried out within an experimental setting that is intrinsically aligned with both the biomedical focus of our core objective and the DEFT 2020 evaluation framework.
